# Changes in Phenolic Acids and Antioxidant Properties during Baking of Bread and Muffin Made from Blends of Hairless Canary Seed, Wheat, and Corn

**DOI:** 10.3390/antiox11061059

**Published:** 2022-05-26

**Authors:** El-Sayed M. Abdel-Aal, Iwona Rabalski

**Affiliations:** Agriculture and Agri-Food Canada, Guelph Research and Development Centre, 93 Stone Road West, Guelph, ON N1G 5C9, Canada; iwona.rabalski@agr.gc.ca

**Keywords:** hairless canary seed groats, whole-grain baked products, polyphenols, antioxidants

## Abstract

Phenolic acids are the major polyphenols in cereal grains and they undergo changes in their composition and structure during processing. This study investigated changes in phenolic acids and antioxidant properties during baking of bread and muffin made from hairless canary seed (HCS), *Phalaris canariensis* L., alone or in blends with corn and wheat. The changes were monitored after dry mixing, dough/batter formation, and oven baking. Phenolic acids were quantified in products using HPLC and antioxidant activity was based on DPPH, ABTS, and ORAC assays. Eight phenolic acids were primarily present in the bound fraction extracts, while only a few phenolic acids were detected in the free or unbound fraction extracts. Ferulic was the dominant phenolic acid in wheat, corn, and HCS followed by *p*-coumaric acid but the latter was extremely high in HCS compared to wheat and corn. After baking, bound phenolic acids decreased in breads and muffins, while the unbound phenolic acids increased. Dough preparation resulted in about 5–13% reductions in bound ferulic acid in addition to 2–9% after oven baking with a total reduction of about 10–20% subject to bread formulation. On the contrary unbound ferulic acid increased by 48–307% after dough preparation and 138–225% after oven baking with a total increase 273–495%. Similarly, muffin-making process resulted in 26–30% reductions in bound ferulic acid after batter preparation and 4–7% after oven baking with reductions of 34–37% in muffins, while the unbound ferulic acid increased by about 35–105% and 9–29%, respectively, with a total increase 47–116%. The baking process resulted in improved DPPH, ABTS, and ORAC antioxidant activities in breads and muffins despite the initial reductions after dough preparation. In general, baking process resulted in tangible increases in unbound phenolic acids which eventually could improve their bioavailability and bioactivity.

## 1. Introduction

Whole-grain cereal foods are considered good sources of diverse array of bioactive compounds with several health-promoting and disease-preventing attributes. The mechanism behind their health benefits has been associated with antioxidant and protective characteristics of dietary fiber, polyphenols, and other bioactive constituents in the grains [1,2]. Hairless (glabrous) canary seed (HCS) is a true cereal grain with a unique nutritional profile due to its fairly high content of bioactive peptides, phenolic acids, and carotenoids with potential health benefits [3]. HCS is a silica free grain and has granted regulatory approvals in Canada and United States for human consumption [4,5]. This opens up a new food market in addition to the existing small birdseed market. Currently several HCS varieties including yellow and brown seeds are available for food use in Canada, the principal producer and exporter of canary seed worldwide, i.e., 65% of the total world production and 80% of total export [6].

Similar to other cereal grains, the main polyphenols in HCS are phenolic acids with ferulic acid being the dominant phenolic compound [7,8]. It has also been found that germination of HCS substantially increases free, bound, and total phenols by 1042, 120, and 741%, respectively compared to the raw or non-germinated seeds [9]. The main phenolic acid fraction in HCS is the insoluble bound accounting for about 90% of the total phenolic acids followed by soluble bound (7.6%) and soluble free (2.2%) [7]. This profile is quite close to the phenolic acid profile of other cereal grains such as wheat. The average contents of caffeic, *o*-coumaric, and ferulic acid are 102, 37, and 212 mg/kg, in brown-seeded hairless canary seed, respectively and 73, 32, and 154 mg/kg in yellow-seeded varieties [8]. Ferulic acid (4-hydroxy-3-methoxycinnamic acid), the main polyphenol in HCS, has health beneficial effects in animals and humans, e.g., lowering blood glucose in diabetic rats or db/db mice [10,11], inhibiting rat intestinal maltase, sucrose, and α-glucosidase [12], and maintaining oxidative status in healthy individuals [13] and improving plasma markers of inflammation and oxidative stress in adults with elevated high-sensitivity C-reactive protein [14] fed whole-grain purple and regular wheat products.

The transformation of cereal grains into foods involves a sequence of technological operations such as milling, dough preparation, and thermal treatment that affect their physical and chemical structure [15]. Thus, it is significant to probe changes in phenolic compounds to understand their behavior during processing [16] and eventually their anticipated health benefits. Research has shown that esters and glycosides of bound phenolic acids get released from the cell wall of cereal grains when thermally processed, having the potential to transform these compounds into more bioavailable forms [17,18]. Other studies have also shown changes in phenolic acids during baking process [19,20,21]. Since HCS is considered a promising functional food ingredient with potential health benefits [3,22,23] having several food applications in baking industry [24], the current study aimed to investigate the effect of baking on phenolic acids and antioxidant properties in breads and muffins made from blends of HCS, wheat, and corn. In a previous study, we investigated changes in carotenoids in those products during baking and their potential to boost consumption of lutein, the main carotenoid in HCS [25].

## 2. Materials and Methods

### 2.1. Chemicals

Ethanol, hexane, acetonitrile, sodium hydroxide, and hydrochloric acid were purchased from Fisher Scientific (Mississauga, ON, Canada). Suprapur formic acid (FA) was purchased from VWR (Mississauga, ON, Canada). 2,2-Diphenyl-1-picrylhydrazyl (DPPH), 2,2-azino-bis (3-ethylbenzothiazoline-6-sulfonic acid) (ABTS), fluorescein, 2,2′-Azobis (2-methylpropion-amidine) dihydrochloride (AAPH), Trolox, gallic, protocatechuic, *p*-hydroxybenzoic, gentisic, 3-hydroxy-benzoic, vanillic, caffeic, syringic, *p*-coumaric, ferulic, sinapinic, and *o*-coumaric acids were purchased from Sigma (Sigma-Aldrich Canada Ltd., Oakville, ON, Canada). All the chemicals and reagents used in the study are of analytical grade. The nano pure water was obtained from Milli-Q integral water purification system (Millipore (Canada) Ltd., Etobicoke, ON, Canada).

### 2.2. Grains and Flours

Hairless canary seed (yellow variety) was kindly provided by the Canary seed Development Commission of Saskatchewan. Hard red spring wheat was kindly obtained from the University of Saskatchewan (Saskatoon, SK, Canada). Yellow corn flour was purchased from the local market in Guelph, ON, Canada. Hairless canary seeds were milled using the UDY cyclone mill (310-014, UDY Corporation, Fort Collins, CO, USA) into whole-grain flour, while the wheat grains were milled on the Brabender Junior mill (Quadrumat Junior, Brabender Instruments Inc., Duisburg, Germany) into whole-grain flour. The grain samples were stored in a walk-in freezer at −20 °C. The flours were stored in a storage fridge at 4 °C for several days until baking trials and analysis were conducted by expert technicians.

### 2.3. Preparation of Bread and Muffin Products

Bread was chosen as a fermented baked product, while muffin represents as a non-fermented baked food. Three bread formulations were made from wheat and HCS flour blends at ratios of 85/15, 75/25, and 50/50 (*w*/*w*) in addition to 100% wheat flour as a control treatment. The sourdough method was used for bread making according to the approved AACCI method 10-11.01 [26]. Three low-fat muffins were prepared from HCS flour alone or in blend with corn flour at ratio of 1:1 and 1:2 (*w*/*w*). The product ingredients and preparation and quality assessment were described in our previous publication [25]. All baking trials were made at least in triplicate and subsamples were taken at three technological steps, e.g., dry mix, batter or dough and freshly baked muffins or breads for monitoring changes in phenolic acids and antioxidants during baking process.

### 2.4. Analysis of Phenolic Acids and Antioxidants

Approximately 0.25 g of grain flours and products was extracted twice with 5 mL of 80% aqueous ethanol on IKA shaker VXB (IKA Works Inc., Wilmington, NC, USA). The extraction was carried out under a nitrogen gas for 30 min. The extract was centrifuged at 4400 rpm for 10 min. The two ethanol extracts were pooled together into a caped clean tube, purged with nitrogen and kept in a freezer until HPLC analysis. The residual pellet obtained after extracting free phenolic acids was further processed for the analysis of bound phenolic acids. First, the pellet was treated with 15 mL of hexane and shaken for 5 min, then centrifuged at 4400 rpm for 10 min. The hexane supernatant was discarded. Five mL of 2 M NaOH was added to the pellet, then the content was purged with nitrogen gas and continuously stirred on IKA stirrer hot plate for 1 h at 70 °C. The mixture was then cooled down to the ambient temperature, acidified to pH 2 with 2 M HCl and centrifuged at 4400 rpm for 10 min. The acidic supernatant was transferred into a clean separatory funnel. The pellet was washed with 10 mL of nano pure water, then centrifuged at 4400 rpm. The aqueous supernatant was combined with the acidic supernatant and the mixture was extracted three times for 5 min each with 10 mL of ethyl ether and ethyl acetate 1:1 (*v*/*v*) using IKA shaker and then centrifuged at 4400 rpm for 5 min each time. The organic phase was collected, passed through anhydrous sodium sulphate, and dried under a stream of nitrogen. The residue was re-dissolved in 4 mL of nano pure water, then filtered through 0.2 µm GHP syringe filter (Pall) and stored in a freezer until HPLC analysis. The free phenolic acid extract was prepared by evaporating 2 mL of ethanol extract to near dryness and re-dissolved in 0.5 mL nano pure water, then filtered and injected onto the HPLC column.

Phenolic acids were analyzed using the Agilent HPLC 1100 series equipped with DAD detection system. Separation of phenolic acids was done on the Supelcosil column LC 18 cat #58298, 25 cm 4.6 mm diameter and 5 m particle size. The following gradient was applied starting with 100% of 6% formic acid (FA) and 0% of acidified acetonitrile (ACN) with FA at 6%. The gradient was gently changed over 35 min to 82% of 6% formic acid and 18% acidified ACN, then kept for 5 min in 100% acidified ACN, and finally 5 min were allowed to return to the starting conditions. The total run time was 45 min. A mixture of 12 authentic phenolic acid standards including gallic, protocatechuic, *p*-hydroxybenzoic, gentisic, 3-hydroxy-benzoic, vanillic, caffeic, syringic, *p*-coumaric, ferulic, sinapinic and *o*-coumaric acids was used for calibration, identification, and quantification. The detection of phenolic acids was performed at five wavelengths (260, 275, 300, 320, 330 nm) in which each phenolic acid was quantified at its maximum absorption wavelength. A wavelength of 260 nm was chosen for detection and quantification of protocatechuic, *p*-hydroxy-benzoic, and vanillic acids. A wavelength of 275 nm was applied to syringic acid, 320 nm wavelength to caffeic, *p*-coumaric, ferulic, sinapinic. More details about the method optimization is previously reported [27].

Antioxidant properties of free and bound product extracts were evaluated based on three assays including scavenging of the stable 2,2-diphenyl-1-picrylhydrazyl radical (DPPH), radical cation (2,2′-azino-di-[3-ethyl benzthiazolinesulphonate] (ABTS) and peroxyl radical or oxygen radical absorbance capacity (ORAC). The three antioxidant methods were described in a previous study [28]. Trolox (6-hydroxy 2,5,7,8-tet-ramethylchroman-2-carboxylic acid) was used as an antioxidant reference and the scavenging activity was calculated in µmol trolox equivalents/g dry weight.

The change in phenolic acids and antioxidants during baking process were calculated as% difference from their corresponding dry mixes using the following equation:

% difference = [carotenoid in product − carotenoid in flour mix]/[carotenoid in flour mix] × 100

### 2.5. Statistical Analysis

Baking trials and analyses were carried out at least in triplicate and the data are expressed as means ± SD (standard deviation) on a dry matter basis. One-way ANOVA was used to determine the effect of baking process on phenolic acids and antioxidants. Significant differences between means were assessed using Tukey method and considered significant at *p* < 0.05. Pearson correlation analysis was conducted to establish relationships between phenolic acids and antioxidants. Statistical analyses were performed using Sigma-Plot version 14.5 (Systat Software Inc., San Jose, CA, USA).

## 3. Results and Discussion

### 3.1. Phenolic Acid Composition and Antioxidant Properties of Raw Ingredients

Whole-grains are considered good sources of antioxidants and bioactive components such as polyphenols, carotenoids, and many others. Since phenolic acids are the main polyphenols in cereal grains, their composition was investigated and monitored during baking process. Eight phenolic acids were found in the bound fraction, while only two phenolic acids (ferulic and *p*-coumaric) were detected in the free or unbound fraction of hairless canary seed (HCS), wheat, and corn extracts (Table 1). The three grains exhibited a close phenolic acid profile, but significant differences were observed among them in the concentration of phenolic acids. Ferulic was the main soluble phenolic acid in HCS and wheat, while *p*-coumaric acid was the major soluble compound in corn. For the bound fraction, ferulic acid was present at an extremely higher concentration compared to other phenolic acids, followed by *p*-coumaric acid (Table 1). Both ferulic and *p*-coumaric were the two dominant bound phenolic acids in HCS, wheat, and corn making up 94, 96, and 98% of the total bound phenolic acids, respectively. These results are in agreement with earlier studies [7,29]. However, in wheat and corn, bound ferulic acid alone made up 93–94% of the total bound phenolic acids, while it was only 60% in HCS as bound *p*-coumaric acid was present in HCS at higher concentration (34% of the total phenolic acids) than wheat and corn (2 and 5%, respectively). Earlier research reported phenolic acids and flavonoids as the most representative groups of polyphenols in modern and ancient wheat along with others such as coumarins, stilbenes, proanthocyanidins, and lignans [30]. In fact, cereal grains contribute to a good portion of dietary polyphenols in Spain estimated at 360 mg/person/day (65 mg extractable and 295 mg non-extractable) [31] which is comparable to that consumed from fruits and vegetables in France (180–345 mg/person) [32].

The three grains exhibited potent antioxidant activities for their free and bound phenolic extracts with significant differences among them (Table 1). These differences are attributed to the differences in their content of phenolic acids (Table 1) and flavonoids [33]. Interestingly, the free phenolic extracts exhibited higher DPPH and ABTS scavenging activities compared to their corresponding bound phenolic extracts for the three grains except for DPPH assay in wheat. The free extracts contain other antioxidant components such as reducing sugars, amino acids, and proteins in addition to the free phenolic acids which could contribute to the DPPH and ABTS scavenging activities. For ORAC assay, bound phenolic acids had substantially higher antioxidant activities than their corresponding free phenolic extracts. This could be attributed to the ability of phenolic acids to preferably scavenge peroxyl radicals. A study has shown significant differences in phenolic acids and DPPH scavenging activity among 25 cereal grains with great antioxidant potential [34]. The differences in phenolic acids, antioxidant activity, and other bioactive compounds among the three cereals would make them suitable ingredients for blending in order to make nutritious foods with potential health benefits.

### 3.2. Changes in Phenolic Acids and Antioxidants in Breads

Bread is a staple food which is consumed often every day in many parts of the world. Indeed, it can be considered a suitable vehicle for many nutrients and health-enhancing components. In a previous study, HCS is added to bread formulations to enhance its content of carotenoids [25]. HCS could also complement other bioactive compounds in wheat such as polyphenols and bioactive peptides. In the current study, three bread formulations made from wheat/HCS at ratio of 85/15, 75/25, and 50/50 (*w*/*w*) were evaluated to probe the behavior of phenolic acids during the bread-making process in comparison with a control bread (100% wheat). Quality and nutrient contents of these products has been previously reported [25]. Significant changes in free and bound phenolic acids occurred during dough formation and oven baking (Table 2).

Despite there was a slight increase in soluble *p*-coumaric acid (CA), there were no significant differences found among the three composite and control flour breads possibly due to its small concentration in the products. However, significant changes occurred in free ferulic acid (FA) during dough preparation and oven baking of breads possibly due to its higher concentration compared to the free CA. There was an increase in free FA by about 236–307% and 138–188% due to dough preparation and oven baking, respectively in the composite flour breads compared to 48% and 273% in the control flour bread. The total increase in free or unbound FA in the end products was 374–495% for the composite flour breads compared to 273% for the control bread. These results are supported by the reduction in bound FA at levels of 4.6–13.1% and 2.0–9.0% for the composite flour breads due to dough formation and oven baking, respectively compared to 5.5% and 9.4% for the control bread. The degree of FA change was dependent on the replacement level of HCS in the composite flours. Similar trends have been reported for breads made from whole-grain einkorn wheat in which free FA increases by 32% while bound FA decreases by 38% [27]. Significant increases in free phenolic acids have also been found in breads baked from wheat, spelt, and rye with the highest increase in rye bread [18]. In addition, the type of fermentation (e.g., yeast versus bacteria) affects phenolic acids differently. Another study reported a significant increase in free FA in purple wheat dough after mixing and fermentation [21]. Phenolic acids are primarily present in cereal grains in the bound form esterified or ether-linked to polysaccharides to cross-link them in the cell wall of the grain to form shielding networks [35]. This structure breaks down during processing resulting in the release of phenolic acids [17,19]. In the current study it appears that dough preparation (mixing and fermentation) induces more changes in phenolic acids than thermal or oven baking maybe due to the exposure of phenolic acids to enzymes in flours and during microbial fermentation (yeast or bacteria). Fermentation with different strains of lactic acid bacteria also causes small to large changes in the distribution of free, soluble conjugated, and insoluble-bound phenolic acids in wheat bread doughs subject to strain type [36]. In another study yeast fermentation had little effect on phenolic acids in doughs made from refined or whole-grain wheat flour [20]. These results substantiate the hypothesis that dough preparation facilitate the release of bound phenolic acids into free forms. The increase in free FA in bread products could improve its bioavailability and eventually the anticipated health beneficial effects. FA has been found to be the main metabolite in serum of persons fed whole-grain wheat products [13,37] and to improve plasma markers of inflammation and oxidative stress [14]. In general, the baking process resulted in substantial increases in free FA indicating a potential for improved bioavailability.

Dietary antioxidants play significant roles in promoting human health and reducing chronic diseases [38]. In this regard, cereal whole-grain foods are considered good sources of dietary antioxidants including phenolic compounds. In the current study, changes in antioxidant activity during baking process were assessed based on three assays due to the variety of oxidants and radicals present in cereal foods [28]. The DPPH scavenging activity of free and bound phenolic extracts of doughs and breads were significantly different compared to their corresponding dry flour mixes (Table 3). 

In addition, incorporation of HCS resulted in slight reductions in DPPH scavenging ability for free and bound phenolic factions of composite flour breads in comparison with the control bread (e.g., 2.8 vs. 2.9 µmol trolox equivalent/g). Dough preparation resulted in reductions in DPPH scavenging activity of free phenolic extracts by 6.5–41.7% subject to bread formulation, while oven baking enhanced DPPH scavenging activity of free phenolic extracts (12.9–58.4%). The reduction in DPPH scavenging activity of doughs could be due to the loss of free sugars and soluble proteins which contribute to the scavenging capacity in the DPPH assay. Whereas the increase in the DPPH scavenging activity of breads could be attributed to Maillard reaction products occurring during oven baking [39]. For bound phenolic extracts, dough preparation and oven baking resulted in an increase in DPPH scavenging capacity. The ABTS scavenging activity was also significantly influenced by the baking process (Table 3). Similar to DPPH results, there were reductions in ABTS scavenging activity of free phenolic extracts after dough preparation (15.9–27.4%) and oven baking (8.2–21.6%), while ABTS scavenging activity of bound phenolic extracts increased after dough preparation by 1.8–29.1% and oven baking by 2.5–25.0%. This indicates differences between free and bound extract in their contents as free extracts contain other non-phenolic antioxidants. ORAC values were also significantly affected by baking process (Table 3). The ability of free phenolic extracts to scavenge peroxyl radicals decreased by 12.6% in the control bread treatment and 1.0% in the W/HCS 85/15 treatment, while it slightly increased in W/HCS 75/25 (0.9%) and W/HCS 50/50 (4.9%) treatments after dough preparation. However, oven baking increased scavenging of peroxyl radicals by free phenolic extracts possibly due to the liberation of more free phenolic compounds and Maillard reaction products. Dough preparation and oven baking slightly increased scavenging of peroxyl radicals by bound phenolic acids. Overall, breads had higher ORAC values than flours and doughs for both free and bound phenolic fractions. Previous research has shown that baking process significantly increases antioxidant activity of breads made from purple wheat [21] or rye [18]. Overall, the baking process resulted in improved antioxidant activity for breads made from blends of wheat and HCS despite the initial reduction during dough preparation.

### 3.3. Changes in Phenolic Acids and Antioxidants in Muffins

Three low-fat muffin products were made from HCS alone or in blends with corn (HCS/corn 1:1 and 1:2, *w*/*w*) to study the effect of baking process on phenolic acids and antioxidant properties. The HCS muffins are highly acceptable and considered a good source of carotenoids [25]. Corn was chosen with HCS to improve the appearance and nutritional profile of muffins, especially their content of carotenoids (e.g., lutein and zeaxanthin). Blending HCS and other underutilized grains (e.g., millet, quinoa, sorghum) with corn at replacement level of 25% has been found to improve their nutritional profile (e.g., increase protein, dietary fiber and ash, while reduce lipids and carbohydrates) resulting in better extruded products compared with corn alone [40]. Table 4 shows the composition of free and bound phenolic acids in muffins made from HCS alone or in blends with corn, and the impact of baking process on phenolic acids. The muffin-making process significantly altered the content of free and bound phenolic acids in muffin products. Similar to bread products, the preparation of batter and oven baking significantly increased the content of free *p*-coumaric and ferulic acid in muffin products. The addition of corn resulted in an increase in free *p*-coumaric acid and reduction in free ferulic acid due to the higher content of bound *p*-coumaric in HCS compared to corn (109.2 vs. 13.4 µg/g), and the lower content of bound ferulic acid (194 vs. 262 µg/g). On the contrary, bound *p*-coumaric and ferulic acid decreased in the muffin products. The total reduction for each bound phenolic acid was fairly close among the three muffin products, e.g., 32–42% and 34–37% for *p*-coumaric and ferulic acid, respectively. In general, the muffin-making process increased total free phenolic acids by about 100% in HCS muffin compared to 64–66% in HCS/corn muffins, while total bound phenolic acids decreased by about 35–36% in the three muffin products. Previous research has reported similar results, i.e., increases in free phenolic acids and reductions in bound phenolic acids in muffins made from whole-grain einkorn wheat [27]. In addition, the extent of increase or reduction was different between bread and muffin products due to differences in their ingredients and baking conditions.

Similar to bread products, the muffin-making process resulted in significant changes in DPPH, ABTS, and ORAC antioxidant values (Table 5). The batter preparation resulted in reductions in the ability of free and bound phenolic acid fractions to scavenge DPPH, ABTS, and peroxyl radicals except for scavenging of peroxyl radical by the free phenolic fraction which increased after batter preparation. On the other hand, oven baking caused improving effects on DPPH, ABTS, and ORAC antioxidant activities of free and phenolic acid fractions. Generally, the overall effect of muffing-making process resulted in increases in DPPH, ABTS, and ORAC antioxidant activities to different extents subject to the type of phenolic fraction, type of assay, and type of formulation. The presence of fat in batter could minimize the availability of antioxidants resulting in lowering their antioxidant activity, while Maillard reaction products occurring during oven baking could contribute to the overall antioxidant activity in muffins. Both products, bread and muffin, are considered good sources of antioxidants when their DPPH, ABTS, and ORAC are compared with pure antioxidants such as ferulic acid, *p*-coumaric acid, caffeic acid, lutein, tocopherols, vitamin C, etc., [28,41]. 

### 3.4. Relationships between Phenolic Acids and Antioxidants

Phenolic acids in foods are considered potent antioxidants due to their ability to function as free radical scavengers and/or reductants. The correlation between phenolic acids and antioxidant activities in breads, muffins and all products combined exhibited different relationships subject to the type of radical (e.g., antioxidant assay) and type of phenolic fraction (Table 6). For the DPPH assay of free phenolic acids, insignificant correlations were observed between DPPH scavenging activity and any of the phenolic acid investigated either in bread or in muffin products. This finding could indicate a little contribution of free phenolic acids to DPPH scavenging capacity due to the presence of other antioxidants in free ethanol extracts such as reducing sugars, vitamins, amino acids, and proteins. Sugars [42], vitamins [43], and proteins [44] are able to scavenge DPPH radicals. Bound phenolic acids also had insignificant correlation with scavenging of DPPH radical except for the total bound phenolic acids in breads perhaps due to the small sample size. When the two products were combined, bound *p*-coumaric acid, ferulic acid, and total bound phenolic acids were significantly correlated with DPPH radical scavenging activity due to the lager sample size (*n* = 21). This shows the ability of phenolic acids to scavenge DPPH radical. Phenolic acids exhibit more than 85% radical scavenging capacity due to the high reactivity of the hydroxyl radical with cinnamic acid derivatives (e.g., ferulic and *p*-coumaric acids) being better scavengers than benzoic acid derivatives (e.g., vanillic and protocatechuic acids) [45].

A slightly different trend was observed for the relationship between ABTS radical scavenging capacity and phenolic acids. There were insignificant correlations between unbound *p*-coumaric or ferulic acid and ABTS scavenging capacity except for unbound *p*-coumaric in breads (*p* = 0.049) (Table 6). The combined data of the two products, however, showed significant correlations with free *p*-coumaric (*p* = 0.00021) and free ferulic acid (*p* = 0.00119) but the direction of relationship is different. Thus, the overall correlation between ABTS radical scavenging capacity and total unbound phenolic acids was insignificant. This unpredicted effect could be due to the presence of non-phenolic antioxidants in the ethanol extracts. The bound *p*-coumaric and ferulic acids had insignificant correlations with ABTS radical scavenging capacity in breads. In muffins, bound *p*-coumaric acid and total bounds significantly correlated with ABTS radical scavenging capacity (*p* = 0.00104 and 0.0269, respectively). Differences between breads and muffins indicate that the food matrix also influences the ability of foods to scavenge free radical. The combined data showed significant contributions of bound phenolic acids to ABTS radical scavenging, e.g., *p*-values for bound ferulic acid and total bounds were 0.00006 and 00003, respectively.

In ORAC assay, there were significant correlations with unbound *p*-coumaric acid in breads (*p* = 0.00031) and muffins (*p* = 0.0326) but not in the case of combined data (*p* = 0.0563) (Table 6). This indicates the relatively powerful scavenging capacity of phenolic acids toward peroxyl radical compared with other non-phenolic antioxidants present in the ethanol extracts due to their high reactivity via hydrogen atom transfer [45]. The unbound ferulic acid, however, exhibited insignificant correlations with ORAC values either in breads or in muffins. The total unbound phenolic acids had significant correlations with ORAC values in muffins and combined data, but insignificant correlations in breads. The bound *p*-coumaric acid showed significant correlations with ORAC values in muffins and insignificant associations in breads and vice versa for bound ferulic acid, i.e., significant correlations in breads and insignificant associations in muffins. Once again this indicates the effect of food type and matrix structure on antioxidant properties. It appears that unbound and bound phenolic extracts from bread and muffin products contribute to their antioxidant properties but several factors control their ability to scavenge free radicals such as their exposure to oxidants, interactions with other antioxidants, type of phenolic acid, and food type.

## 4. Conclusions

Blending of cereal grains holds a promise in making functional ingredients due to their differences in bioactive compounds and antioxidant properties which could complement each other resulting in nutritious foods with potential health benefits. In this regard, hairless canary seed, a true cereal grain rich in protein, bioactive peptides, and carotenoids, has the potential to be a functional food ingredient in several baking and snack formulations. In the current study, bread and muffin made from hairless canary seed alone or in blends with corn and wheat were developed and evaluated based on their content of phenolic acids and antioxidant properties and their changes during baking process. In a previous study, the bread and muffin products were assessed in terms of their content of carotenoids and the impact of baking on carotenoid composition [25]. The current study complements the carotenoid study as it investigates another group of bioactive compounds or polyphenols and antioxidant properties. Ferulic and *p*-coumaric acids were the dominant phenolic compounds in breads and muffins primarily in the bound form. The baking process resulted in reductions in bound phenolic acids, while the unbound phenolic acids increased. The DPPH, ABTS, and ORAC antioxidant activities improved in breads and muffins despite the initial reduction after dough preparation. In general, baking process resulted in tangible increases in unbound phenolic acids which eventually could improve their bioavailability. The present study provides new information on HCS food products and their potential to boost the daily intake of polyphenols and carotenoids as proved health-enhancing components. Further research is underway to assess bioavailability of phenolic acids and carotenoids in muffins and breads made from hairless canary seed in blends with wheat and corn.

## Figures and Tables

**Table 1:** Composition of unbound and bound phenolic acids from hairless canary seed, wheat, and corn raw ingredients and their antioxidant properties (mean ± SD) ^x^.

Phenolic Acid	Hairless Canary Seed	Wheat	Corn
Unbound fraction (µg/g)			
*p*-Coumaric	0.63 ± 0.03 ^b^	0.13 ± 0.01 ^c^	1.77 ± 0.11 ^a^
Ferulic	0.91 ± 0.04 ^b^	1.43 ± 0.06 ^a^	0.11 ± 0.0 ^c^
Total unbounds	1.54	1.56	1.88
Bound fraction (µg/g)			
Protocatechuic	2.79 ± 0.12 ^a^	0.12 ± 0.01 ^b^	0.17 ± 0.01 ^b^
*p*-Hydroxy-benzoic	8.48 ± 0.16 ^a^	2.06 ± 0.04 ^b^	1.18 ± 0.06 ^c^
Vanillic	6.82 ± 0.06 ^a^	6.85 ± 0.31 ^a^	1.69 ± 0.07 ^b^
Caffeic	0.52 ± 0.03 ^a^	0.23 ± 0.01 ^b^	0.27 ± 0.01 ^b^
Syringic	0.81 ± 0.01 ^c^	4.48 ± 0.20 ^a^	2.05 ± 0.11 ^b^
*p*-Coumaric	109.2 ± 0.60 ^a^	8.13 ± 0.32 ^b^	13.36 ± 0.61 ^b^
Ferulic	193.9 ± 6.14 ^c^	354.2 ± 2.58 ^a^	261.5 ± 4.57 ^b^
Sinapic	1.21 ± 0.02 ^a^	1.51 ± 0.03 ^a^	0.33 ± 0.02 ^b^
Total bounds	323.7	377.6	280.6
Antioxidant capacity (µmol TE/g) **			
DPPH, unbound fraction	2.68 ± 0.04 ^a^	2.32 ± 0.11 ^b^	2.65 ± 0.13 ^a^
DPPH, bound fraction	2.07 ± 0.03 ^b^	2.64 ± 0.05 ^a^	2.27 ± 0.04 ^b^
ABTS, unbound fraction	10.12 ± 0.17 ^b^	10.03 ± 0.42 ^b^	12.34 ± 0.39 ^a^
ABTS, bound fraction	4.61 ± 0.11 ^b^	6.45 ± 0.15 ^a^	3.27 ± 0.16 ^c^
ORAC, unbound fraction	16.8 ± 0.27 ^b^	15.11 ± 0.19 ^b^	18.49 ± 0.68 ^a^
ORAC, bound fraction	101.1 ± 1.05 ^a^	98.4 ± 0.60 ^b^	66.8 ± 0.95 ^c^

^x^ Means in a row followed by a different superscript letter are significantly different at *p* < 0.05. ** TE, Trolox Equivalent.

**Table 2:** Composition of unbound and bound phenolic acids of dry flour mix, dough, and fresh bread products and the impact of baking process on their concentrations ^x^.

Phenolic Acid	Wheat (100%)			Wheat/Hairless Canary Seed (85/15, *w*/*w*)			Wheat/Hairless Canary Seed (75/25, *w*/*w*)			Wheat/Hairless Canary Seed (50/50, *w*/*w*)		
	Flour Mix	Dough	Bread	Flour Mix	Dough	Bread	Flour Mix	Dough	Bread	Flour Mix	Dough	Bread
Unbound fraction (µg/g)												
*p*-Coumaric	0.11 ± 0.01 ^a^	0.13 ± 0.01 ^a^	0.14 ± 0.01 ^a^	0.15 ± 0.01 ^a^	0.18 ± 0.01 ^a^	0.18 ± 0.01 ^a^	0.15 ± 0.01 ^a^	0.17 ± 0.01 ^a^	0.18 ± 0.01 ^a^	0.21 ± 0.01 ^a^	0.23 ± 0.01 ^a^	0.24 ± 0.01 ^a^
Ferulic	1.53 ± 0.06 ^a^	2.26 ± 0.03 ^b^	5.70 ± 0.20 ^c^	1.39 ± 0.06 ^a^	4.67 ± 0.60 ^b^	6.59 ± 0.18 ^c^	1.27 ± 0.03 ^a^	4.46 ± 0.24 ^b^	6.62 ± 0.17 ^c^	0.95 ± 0.04 ^a^	3.87 ± 0.20 ^b^	5.65 ± 0.20 ^c^
Total unbounds	1.64	2.39	5.84	1.54	4.85	6.77	1.42	4.63	6.80	1.16	4.10	5.89
Bound fraction (µg/g)												
*p*-hydroxy-benzoic	3.42 ± 0.06 ^a^	3.11 ± 0.05 ^b^	3.01 ± 0.05 ^b^	6.03 ± 0.34 ^a^	4.28 ± 0.11 ^b^	3.33 ± 0.19 ^c^	6.14 ± 0.31 ^a^	5.18 ± 0.22 ^b^	4.34 ± 0.08 ^c^	6.72 ± 0.39 ^a^	5.68 ± 0.29 ^b^	5.71 ± 0.15 ^b^
Vanillic	5.95 ± 0.24 ^a^	5.51 ± 0.13 ^b^	3.49 ± 0.16 ^c^	7.54 ± 0.31 ^a^	6.67 ± 0.23 ^b^	5.80 ± 0.21 ^c^	7.22 ± 0.05 ^a^	6.56 ± 0.25 ^b^	5.85 ± 0.14 ^c^	7.35 ± 0.27 ^a^	6.58 ± 0.29 ^b^	5.75 ± 0.23 ^c^
Caffeic	0.15 ± 0.01 ^a^	0.13 ± 0.02 ^a^	0.11 ± 0.01 ^a^	0.25 ± 0.01 ^a^	0.26 ± 0.02 ^a^	0.21 ± 0.01 ^c\ a^	0.26 ± 0.01 ^a^	0.27 ± 0.02 ^a^	0.26 ± 0.01 ^a^	0.30 ± 0.02 ^a^	0.28 ± 0.02 ^a^	0.25 ± 0.01 ^a^
Syringic	4.58 ± 0.13 ^a^	3.97 ± 0.20 ^b^	2.91 ± 0.14 ^c^	4.39 ± 0.19 ^a^	3.59 ± 0.13 ^b^	2.35 ± 0.14 ^c^	4.09 ± 0.16 ^a^	3.53 ± 0.12 ^b^	2.59 ± 0.11 ^c^	3.86 ± 0.17 ^a^	3.13 ± 0.12 ^b^	2.27 ± 0.11 ^c^
*p*-Coumaric	9.23 ± 0.46 ^a^	9.18 ± 0.23 ^a^	8.35 ± 0.22 ^b^	30.10 ± 1.26 ^a^	25.46 ± 1.22 ^b^	24.38 ± 1.07 ^c^	32.70 ± 0.35 ^a^	30.77 ± 1.27 ^b^	24.47 ± 1.06 ^c^	54.9 ± 0.55 ^a^	53.3 ± 2.27 ^b^	51.1 ± 1.24 ^c^
Ferulic	343.7 ± 17.11 ^a^	324.9 ± 5.13 ^b^	292.5 ± 1.63 ^c^	325.5 ± 7.67 ^a^	310.5 ± 12.35 ^b^	281.3 ± 11.60 ^c^	308.9 ± 14.06 ^a^	283.2 ± 12.64 ^b^	277.2 ± 12.71 ^c^	294.8 ± 14.17 ^a^	255.5 ± 7.26 ^b^	234.7 ± 12.68 ^c^
Sinapic	1.53 ± 0.06 ^a^	1.46 ± 0.03 ^b^	1.27 ± 0.03 ^c^	1.32 ± 0.05 ^a^	1.25 ± 0.03 ^b^	1.07 ± 0.03 ^c^	1.22 ± 0.06 ^a^	1.13 ± 0.03 ^b^	0.97 ± 0.03 ^c^	1.07 ± 0.04 ^a^	1.01 ± 0.03 ^a^	0.87 ± 0.02 ^b^
Total bounds	369	348	312	376	352	318	361	331	316	369	326	301
% Difference (+, increase and −, decrease)												
Unbound *p*-coumaric	-	18.2	27.3 (9.1) ^y^	-	20.0	20.0 (0.0)	-	13.3	20.0 (6.7)	-	9.5	14.3 (4.8)
Bound *p*-coumaric	-	−0.5	−9.5 (−9.0)	-	−15.4	−19.0 (−3.6)	-	−5.9	−25.2 (−19.3)	-	−2.9	−6.9 (−4.0)
Unbound ferulic acid	-	47.7	273 (225)	-	236	374 (138)	-	251	421 (170)	-	307	495 (188)
Bound ferulic acid	-	−5.5	−14.9 (−9.4)	-	−4.6	−13.6 (−9.0)	-	−8.3	−10.3 (−2.0)	-	−13.1	−20.4 (−7.1)
Total unbounds	-	45.7	256 (210)	-	215	340 (125)	-	226	379 (153)	-	253	408 (155)
Total bounds	-	−5.7	−15.4 (−9.8)	-	−6.4	−15.4 (−9.0)	-	−8.3	−12.5 (−4.2)	-	−11.7	−18.4 (−6.7)

^x^ For each product and phenolic acid, means in a row followed by a different superscript letter are significantly different at *p* < 0.05. ^y^ Figures in parenthesis are reduction % due to oven baking.

**Table 3:** Antioxidant properties of unbound and bound phenolic extracts of dry flour mix, dough, and fresh bread products and their influence by baking process ^x^.

Antioxidant Capacity (µmol TE/g)	Wheat (100%)			Wheat/Hairless Canary Seed (85/15, *w*/*w*)			Wheat/Hairless Canary Seed (75/25, *w*/*w*)			Wheat/Hairless Canary Seed (50/50, *w*/*w*)		
	Flour Mix	Dough	Bread	Flour Mix	Dough	Bread	Flour Mix	Dough	Bread	Flour Mix	Dough	Bread
DPPH, unbound fraction	3.1 ± 0.06 ^a^	2.5 ± 0.09 ^b^	2.9 ± 0.10 ^c^	2.4 ± 0.07 ^a^	2.1 ± 0.04 ^b^	2.7 ± 0.11 ^c^	2.4 ± 0.09 ^a^	1.4 ± 0.03 ^b^	2.8 ± 0.05 ^c^	2.4 ± 0.12 ^a^	1.8 ± 0.04 ^b^	2.8 ± 0.10 ^c^
DPPH, bound fraction	2.9 ± 0.09 ^a^	3.2 ± 0.13 ^b^	3.3 ± 0.08 ^c^	2.6 ± 0.10 ^a^	3.2 ± 0.14 ^b^	3.3 ± 0.12 ^c^	2.4 ± 0.10 ^a^	3.4 ± 0.16 ^b^	3.2 ± 0.12 ^c^	2.1 ± 0.07 ^a^	3.0 ± 0.13 ^b^	2.8 ± 0.14 ^c^
ABTS, unbound fraction	5.1 ± 0.17 ^a^	3.8 ± 0.09 ^b^	2.7 ± 0.10 ^c^	7.3 ± 0.22 ^a^	5.3 ± 0.19 ^b^	4.7 ± 0.11 ^c^	8.8 ± 0.14 ^a^	7.4 ± 0.20 ^b^	6.3 ± 0.10 ^c^	10.2 ± 0.30 ^a^	8.5 ± 0.22 ^b^	7.6 ± 0.21 ^c^
ABTS, bound fraction	5.64 ± 0.26 ^b^	5.72 ± 0.16 ^b^	7.11 ± 0.34 ^a^	5.51 ± 0.22 ^b^	7.10 ± 0.33 ^a^	7.24 ± 0.19 ^a^	5.27 ± 0.19 ^b^	6.11 ± 0.23 ^a^	6.28 ± 0.26 ^a^	5.15 ± 0.20 ^b^	5.89 ± 0.21 ^a^	6.05 ± 0.24 ^a^
ORAC, unbound fraction	17.4 ± 0.75 ^a^	15.2 ± 0.39 ^b^	21.4 ± 1.01 ^c^	19.1 ± 0.96 ^a^	18.9 ± 0.89 ^b^	23.6 ± 1.11 ^c^	21.5 ± 1.07 ^a^	21.7 ± 0.60 ^b^	24.7 ± 1.21 ^c^	24.5 ± 1.13 ^a^	25.7 ± 1.16 ^b^	27.9 ± 1.26 ^c^
ORAC, bound fraction	110 ± 5.09 ^a^	117 ± 4.32 ^b^	118 ± 4.29 ^c^	112 ± 5.17 ^a^	121 ± 4.98 ^b^	125 ± 6.21 ^c^	115 ± 4.28 ^a^	124 ± 5.03 ^b^	126 ± 6.11 ^c^	116 ± 4.18 ^a^	125 ± 5.34 ^b^	127 ± 5.47 ^c^
% Difference (+, increase and −, decrease)												
DPPH, unbound fraction	-	−19.4	−6.5 (12.9) ^y^	-	−12.5	12.5 (25.0)	-	−41.7	16.7 (58.4)	-	−25.0	16.7 (41.7)
DPPH, bound fraction	-	10.3	13.8 (3.5)	-	23.1	26.9 (3.8)	-	41.7	33.3 (−8.3)	-	42.9	33.3 (−9.5)
ABTS, unbound fraction	-	−25.5	−47.1 (−21.6)	-	−27.4	−35.6 (−8.2)	-	−15.9	−28.4 (−12.5)	-	−16.7	−25.5 (−8.8)
ABTS, bound fraction	-	1.8	26.8 (25.0)	-	29.1	31.6 (2.5)	-	15.3	18.9 (3.6)	-	13.5	17.3 (3.8)
ORAC, unbound fraction	-	−12.6	23.0 (35.6)	-	−1.0	23.6 (24.6)	-	0.9	14.9 (14.0)	-	4.9	13.9 (9.0)
ORAC, bound fraction	-	6.4	7.3 (0.9)	-	8.0	11.6 (3.6)	-	7.8	9.6 (1.8)	-	7.8	9.5 (1.7)

^x^ For each product and fraction, means in a row followed by a different superscript letter are significantly different at *p* < 0.05. ^y^ Figures in parenthesis are reduction % due to oven baking.

**Table 4:** Composition of unbound and bound phenolic acids of dry flour mix, batter, and fresh muffin products and the impact of baking process on their concentrations ^x^.

Phenolic Acid	Hairless Canary Seed (100%)			Hairless Canary Seed/Corn Blend (1:1, *w*/*w*)			Hairless Canary Seed/Corn Blend (1:2, *w*/*w*)		
	Flour Mix	Batter	Muffin	Flour Mix	Batter	Muffin	Flour Mix	Batter	Muffin
Unbound fraction (µg/g)									
*p*-Coumaric	1.03 ± 0.03 ^a^	1.85 ± 0.06 ^b^	1.91 ± 0.05 ^b^	2.05 ± 0.01 ^a^	2.76 ± 0.05 ^b^	3.42 ± 0.02 ^c^	2.16 ± 0.02 ^a^	2.87 ± 0.09 ^b^	3.66 ± 0.02 ^c^
Ferulic	0.93 ± 0.05 ^a^	1.91 ± 0.06 ^b^	2.01 ± 0.05 ^b^	0.75 ± 0.03 ^a^	1.01 ± 0.03 ^b^	1.23 ± 0.05 ^c^	0.70 ± 0.02 ^a^	0.97 ± 0.03 ^b^	1.03 ± 0.05 ^b^
Total unbounds	1.96	3.76	3.92	2.80	3.77	4.65	2.86	3.84	4.69
Bound fraction (µg/g)									
Protocatechuic	3.65 ± 0.19 ^a^	2.19 ± 0.13 ^b^	1.90 ± 0.08 ^c^	3.13 ± 0.15 ^a^	2.01 ± 0.09 ^b^	1.90 ± 0.08 ^b^	1.93 ± 0.05 ^a^	1.04 ± 0.03 ^b^	0.82 ± 0.02 ^c^
*p*-hydroxybenzoic	9.23 ± 0.33 ^a^	6.93 ± 0.54 ^b^	6.73 ± 0.23 ^b^	4.91 ± 0.23 ^a^	3.94 ± 0.14 ^b^	3.82 ± 0.09 ^b^	3.67 ± 0.13 ^a^	2.39 ± 0.07 ^b^	2.08 ± 0.05 ^b^
Vanillic	8.09 ± 0.39 ^a^	7.32 ± 0.42 ^b^	7.13 ± 0.37 ^b^	4.13 ± 0.16 ^a^	2.42 ± 0.02 ^b^	2.22 ± 0.03 ^b^	4.01 ± 0.11 ^a^	2.72 ± 0.02 ^b^	2.27 ± 0.03 ^c^
Caffeic	0.53 ± 0.02 ^a^	0.38 ± 0.01 ^b^	0.27 ± 0.01 ^c^	0.66 ± 0.02 ^a^	0.41 ± 0.01 ^b^	0.28 ± 0.01 ^c^	0.71 ± 0.02 ^a^	0.38 ± 0.01 ^b^	0.31 ± 0.01 ^b^
Syringic	1.29 ± 0.09 ^a^	0.84 ± 0.03 ^b^	0.49 ± 0.01 ^c^	1.67 ± 0.04 ^a^	0.83 ± 0.03 ^b^	0.53 ± 0.02 ^c^	1.72 ± 0.01 ^a^	0.92 ± 0.03 ^b^	0.59 ± 0.02 ^c^
*p*-Coumaric	117.3 ± 3.81 ^a^	90.7 ± 1.62 ^b^	79.9 ± 1.43 ^c^	61.3 ± 1.61 ^a^	44.2 ± 1.89 ^b^	39.9 ± 0.96 ^c^	43.4 ± 2.26 ^a^	28.1 ± 1.38 ^b^	25.1 ± 1.34 ^c^
Ferulic	194.5 ± 3.66 ^a^	136.2 ± 7.47 ^b^	122.1 ± 1.85 ^c^	203.1 ± 5.72 ^a^	142.1 ± 1.39 ^b^	133.2 ± 5.13 ^c^	208.0 ± 9.67 ^a^	153.2 ± 6.40 ^b^	138.0 ± 6.01 ^c^
Sinapic	1.22 ± 0.05 ^a^	0.77 ± 0.02 ^b^	0.57 ± 0.01 ^c^	0.91 ± 0.02 ^a^	0.65 ± 0.02 ^b^	0.47 ± 0.01 ^c^	0.59 ± 0.02 ^a^	0.37 ± 0.01 ^b^	0.26 ± 0.01 ^c^
Total bounds	336	245	219	280	197	182	264	189	169
% Difference (+, increase and −, decrease)									
Unbound *p*-coumaric acid	-	79.6	85.4 (5.8) ^y^	-	34.6	66.8 (32.2)	-	32.9	69.4 (36.5)
Bund *p*-coumaric acid	-	−22.7	−31.9 (−9.2)	-	−27.9	−34.9 (−7.0)	-	−35.3	−42.2 (−6.9)
Unbound ferulic acid	-	105	116 (10.8)	-	34.7	64.0 (29.3)	-	38.6	47.1 (8.6)
Bound ferulic acid	-	−30.0	−37.2 (−7.2)	-	−30.0	−34.4 (−4.4)	-	−26.3	−33.7 (−7.4)
Total unbounds	-	91.8	100 (8.2)	-	34.6	66.1 (31.5)	-	34.4	64.0 (29.7)
Total bounds	-	−27.1	−34.8 (−7.7)	-	−29.6	−35.0 (−5.4)	-	−28.4	−36.0 (−7.6)

^x^ For each product and phenolic acid, means in a row followed by a different superscript letter are significantly different at *p* < 0.05. ^y^ Figures in parenthesis are reduction % due to oven baking.

**Table 5:** Antioxidant properties of unbound and bound phenolic extracts of dry flour mix, batter, and fresh muffin products and their influence by baking process ^x^.

Antioxidant Capacity (µmol TE/g)	Hairless Canary Seed (100%)			Hairless Canary Seed/Corn Blend (1:1, *w*/*w*)			Hairless Canary Seed/Corn Blend (1:2, *w*/*w*)		
	Flour Mix	Batter	Muffin	Flour Mix	Batter	Muffin	Flour Mix	Batter	Muffin
DPPH, unbound fraction	2.7 ± 0.11 ^a^	1.8 ± 0.05 ^c^	2.2 ± 0.06 ^b^	2.6 ± 0.12 ^a^	1.9 ± 0.08 ^b^	2.4 ± 0.11 ^a^	2.6 ± 0.10 ^a^	1.9 ± 0.05 ^b^	2.1 ± 0.04 ^b^
DPPH, bound fraction	1.9 ± 0.07 ^a^	1.5 ± 0.06 ^b^	1.6 ± 0.07 ^b^	1.6 ± 0.05 ^a^	1.5 ± 0.04 ^a^	1.6 ± 0.06 ^a^	1.6 ± 0.05 ^a^	1.5 ± 0.04 ^a^	1.6 ± 0.06 ^a^
ABTS, unbound fraction	10.3 ± 0.21 ^b^	8.7 ± 0.08 ^c^	14.2 ± 0.67 ^a^	12.1 ± 0.62 ^a^	11.4 ± 0.28 ^b^	11.8 ± 0.49 ^b^	12.4 ± 0.26 ^a^	10.1 ± 0.42 ^b^	10.8 ± 0.25 ^b^
ABTS, bound fraction	4.6 ± 0.21 ^a^	3.6 ± 0.12 ^b^	3.4 ± 0.13 ^b^	3.1 ± 0.14 ^a^	2.6 ± 0.11 ^b^	3.1 ± 0.09 ^a^	2.9 ± 0.12 ^a^	2.2 ± 0.07 ^b^	3.0 ± 0.11 ^a^
ORAC, unbound fraction	15.7 ± 0.84 ^c^	21.8 ± 0.75 ^b^	30.6 ± 1.50 ^a^	16.8 ± 1.04 ^c^	22.1 ± 1.06 ^b^	31.1 ± 1.14 ^a^	18.2 ± 1.44 ^c^	25.1 ± 2.02 ^b^	31.3 ± 1.29 ^a^
ORAC, bound fraction	117 ± 4.18 ^a^	84 ± 3.71 ^c^	87 ± 3.59 ^b^	78 ± 2.22 ^a^	69 ± 2.78 ^b^	71 ± 3.12 ^b^	56 ± 2.09 ^b^	55 ± 2.78 ^b^	61 ± 2.52 ^a^
% Difference (+, increase and −, decrease)									
DPPH, unbound fraction	-	−33.3	−18.5 (14.8) ^y^	-	−26.9	−7.7 (19.2)	-	−26.9	−19.2 (7.7)
DPPH, bound fraction	-	−21.1	−15.8 (5.3)	-	−6.3	0.0 (6.3)	-	−6.3	0.0 (6.3)
ABTS, unbound fraction	-	−15.5	37.9 (53.4)	-	−5.8	−2.5 (3.3)	-	−18.5	−12.9 (5.6)
ABTS, bound fraction	-	−21.7	−26.1 (−4.4)	-	−16.1	0.0 (16.1)	-	−24.9	2.4 (27.3)
ORAC, unbound fraction	-	38.9	94.9 (56.0)	-	31.5	85.1 (53.6)	-	37.9	72.0 (34.1)
ORAC, bound fraction	-	−28.2	−25.6 (2.6)	-	−11.5	−9.0 (2.5)	-	−1.8	8.9 (10.7)

^x^ For each product and fraction, means in a row followed by a different superscript letter are significantly different at *p* < 0.05. ^y^ Figures in parenthesis are reduction % due to oven baking.

**Table 6:** Relationships between phenolic acids and antioxidant activity of bread and muffin products.

Phenolic Acid	DPPH, Unbound	DPPH, Bound	ABTS, Unbound	ABTS, Bound	ORAC, Unbound	ORAC, Bound
Bread (*n* = 12)						
*p*-Coumaric, unbound	−0.314 (0.321) ^ns^	-	0.578 (0.0490) *	-	0.862 (0.00031) ***	-
Ferulic, unbound	0.084 (0.795) ^ns^	-	−0.417 (0.178) ^ns^	-	0.466 (0.126) ^ns^	-
Total unbounds	0.078 (0.810) ^ns^	-	−0.404 (0.193) ^ns^	-	0.479 (0.115) ^ns^	-
*p*-Coumaric, bound	-	−0.548 (0.0652) ^ns^	-	−0.340 (0.279) ^ns^	-	0.378 (0.226) ^ns^
Ferulic, bound	-	−0.128 (0.692) ^ns^	-	−0.264 (0.407) ^ns^	-	−0.872 (0.00022) ***
Total bounds	-	−0.590 (0.0435) *	-	−0.621 (0.0310) *	-	−0.848 (0.00049) ***
Muffin (*n* = 9)						
*p*-Coumaric, unbound	−0.383 (0.310) ^ns^	-	0.0176 (0.964) ^ns^	-	0.709 (0.0326) *	-
Ferulic, unbound	−0.491 (0.180) ^ns^	-	0.0372 (0.924) ^ns^	-	0.476 (0.195) ^ns^	-
Total unbounds	−0.618 (0.0761) ^ns^	-	0.0362 (0.926) ^ns^	-	0.915 (0.00054) ***	-
*p*-Coumaric, bound	-	0.618 (0.0759) ^ns^	-	0.897 (0.00104) **	-	0.940 (0.00016) ***
Ferulic, bound	-	0.465 (0.207) ^ns^	-	0.204 (0.599) ^ns^	-	0.127 (0.744) ^ns^
Total bounds	-	−0.627 (0.0705) ^ns^	-	0.726 (0.0269) *	-	0.708 (0.0327) *
All products (*n* = 21)						
*p*-Coumaric, unbound	−0.297 (0.191) ^ns^	-	0.723 (0.00021) ***	-	0.423 (0.0563) ^ns^	-
Ferulic, unbound	0.163 (0.480) ^ns^	-	−0.658 (0.00119) **	-	0.139 (0.548) ^ns^	-
Total unbounds	−0.018 (0.939) ^ns^	-	−0.274 (0.229) ^ns^	-	0.476 (0.0292) *	-
*p*-Coumaric, bound	-	−0.544 (0.0109) *	-	−0.372 (0.0969) ^ns^	-	−0.122 (0.597) ^ns^
Ferulic, bound	-	0.827 (0.000004) ***	-	0.817 (0.000006) ***	-	0.762 (0.00006) ***
Total bounds	-	0.710 (0.00031) ***	-	0.779 (0.00003) ***	-	0.835 (0.000003) ***

Figures in parenthesis are *p* values; ^ns^, not significantly; * significant at *p* < 0.05; ** significant at *p* < 0.01; *** significant at *p* < 0.001.

## Data Availability

The data presented in this study are available in the article.
